# Unraveling sleep challenges in diabetes mellitus: reflections on recent evidence

**DOI:** 10.1097/MS9.0000000000003978

**Published:** 2025-09-29

**Authors:** Kainat Zulfiqar, Ahmad Furqan Anjum, Hermann Yokolo

**Affiliations:** a Department of Internal Medicine, Rawal Medical College Islamabad, Islamabad, Pakistan; b Department of Internal Medicine, Shaikh Khalifa Bin Zayed Al Nahyan Medical and Dental College, Lahore, Pakistan; c Department of Research, Medical Research Circle (MedReC), Goma, DR Congo

*Dear Editor,*

We read with great interest the article by Hamayal *et al* titled “Prevalence and determinants of poor sleep quality among patients of diabetes mellitus: a tertiary hospital-based cross-sectional study”^[1]^. The present study contributes to the knowledge by illuminating a frequently neglected component of diabetes management, the effect of sleep quality on the health outcomes of patients. The authors deserve credit for reporting this significant public health problem. Their study helps us to further appreciate the fact that sleep disturbances in diabetes are multifactorial and the importance of a multifaceted patient approach. Nevertheless, there are some limitations of this work, beyond the limitations stated by the authors themselves, that should be taken into account. This article complies with the TITAN 2025 guidelines – repressing the reporting and use of AI^[2]^.

First, the risk of selection bias occurs as the patients who had severe diabetic complications or cognitive impairment (at least unable to complete the Pittsburgh Sleep Quality Index (PSQI) questionnaire) were excluded. This could contribute to the under-presentation of patients with the poorest quality of sleep.

It is possible that poor sleep is overestimated because the severity of complications may have resulted in an increased number of sleep disturbances. The relationship between the severity of diabetes and poor sleep can be weakened. For example, Luyster *et al* brought out the indication that the exclusion of extremely sick patients may bias prevalence rates of sleep problems^[3]^. The next research on the subject should involve patients with both minor and severe presence of the condition, maybe with the aid of co-carer questionnaires or a simplified form of in-depth sleep systems in people with cognitive or physical disability. Second, no such comparison in terms of a control group (of nondiabetic individuals). It is challenging to estimate whether the high prevalence is actually related only to diabetes, not just the general population trends, unless a baseline is established. For example, there is a large Norwegian study by Siverstsen *et al* that involved both diabetic and nondiabetic patients, which makes it possible to compare insomnia prevalence directly and is precisely about why a control group is significant in the diabetes sleep quality scenario^[4]^.To better establish how diabetes has a negative impact on the quality of sleep, future studies should incorporate a matched but not diabetic control group to shed light on the impact of diabetes on sleep. Third, the use of only self-reported comorbidities and control of glycemic status without verification using medical records. This brings about recall and reporting bias. Such misclassification may dilute or even produce spurious results about predictors of poor sleep. For example, in a research paper by Tisnado *et al*, self-reported health conditions were compared with medical records, where a huge disparity was encountered, which is why unverified self-report has the capacity to skew study findings^[5]^. Future studies should check the comorbidities and the level of HbA1c using hospital records in order to increase accuracy. Fourth, failure to measure the psychological factors that include the level of depression and anxiety, which have been found to be significant in the quality of sleep among diabetic people. Confounders not reported may result in the overestimation of diabetes-specific effects of sleep. For example, the study by Botros *et al* (2009) proves the mediating role of the factors of mental health in the quality of sleep^[6]^. It is recommended to include levels of depression/anxiety using tests (i.e., Patient Health Questionnaire – 9 items (PHQ-9) and Generalized Anxiety Disorder – 7 items (GAD-7)) in future research. Fifth, failure to consider medication effects (medications such as beta-blockers, statins, and antidepressants that could affect sleep) that might disrupt sleep. This may mislead a correlation by ascribing poor sleep to medication-related issues instead of diabetes itself. For example, in an article by Sateia *et al* (2014), there is a list of medications that induce sleep disturbance^[7]^. Therefore, future studies should obtain thorough drug histories and analyze them in accordance with adjustments made to drugs that are known factors influencing sleep.

Conclusively, even though this research offers valuable information about the rate and predictors of having low-quality sleep in patients with diabetes, the drawbacks discussed above must also be considered because they affect the interpretation of the research results. In future studies, these problems will result in more valid, generalizable, and clinically applicable findings, leading to a more robust evidence base to inform the targeted intervention to reduce sleep health in this group.

## Data Availability

Not applicable.
